# Supplementary material for: Construction of the Cellulose Nanofibers (CNFs) Aerogel Loading TiO2 NPs and Its Application in Disposal of Organic Pollutants
Source: Polymers (Basel). 2021 Jun 2;13(11):1841. doi: 10.3390/polym13111841 (PMC8199583; doi:10.3390/polym13111841)
Supplement: Supplementary file 1 [file polymers-13-01841-s001.zip › polymers-1221844-supplementary.pdf]

polymers

MDPI

Supplementary Material: Construction of the Cellulose Nanofibers (CNFs) Aerogel Loading TiO_2 NPs and Its Application in Disposal of Organic Pollutants

Kang Li, Xuejie Zhang, Yan Qin, Ying Li

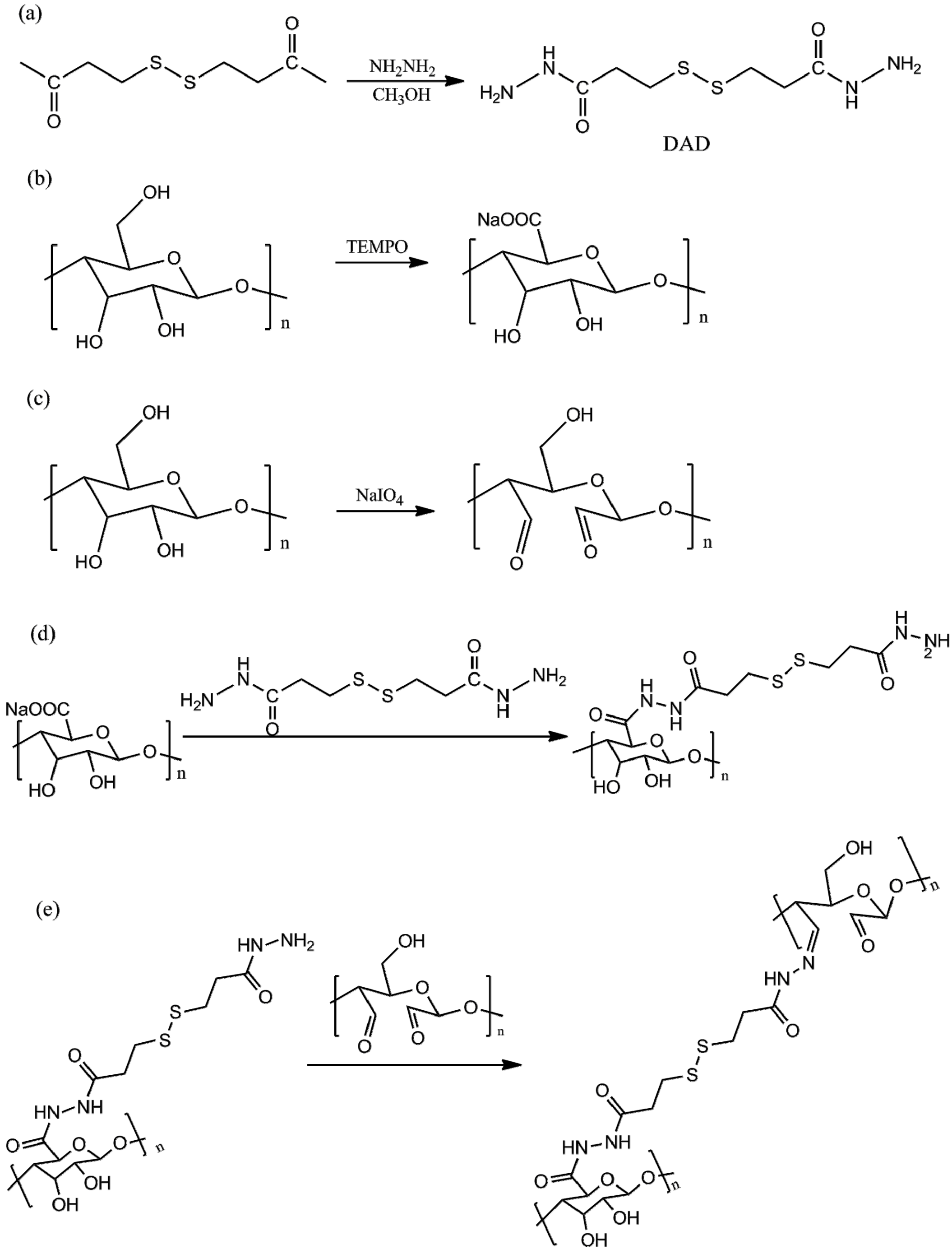

Scheme S1. (**a**) The synthetic route of DAD, (**b**) the synthetic route of CNFs-COONa (**c**) the synthetic route of CNFs-CHO, (**d**) the synthetic route of CNFs-DAD, (**e**) the synthesis route of the CNFs aerogel crosslinked by hydrazone bond.

Table S1. Surface functional group content of CNFs-CHO, CNFs-COONa and CNFs-DAD.

Type of CNFs	Carbonyl content (mmol/g)	Aldehyde content (mmol/g)	Amino content (mmol/g)
CNFs-COONa	1.15	-	-
CNFs-DAD	0.78	-	0.37
CHO-CNFs	-	0.86	-
